# Short-Term Coral Bleaching Is Not Recorded by Skeletal Boron Isotopes

**DOI:** 10.1371/journal.pone.0112011

**Published:** 2014-11-14

**Authors:** Verena Schoepf, Malcolm T. McCulloch, Mark E. Warner, Stephen J. Levas, Yohei Matsui, Matthew D. Aschaffenburg, Andréa G. Grottoli

**Affiliations:** 1 School of Earth and Environment, The University of Western Australia and ARC Centre of Excellence for Coral Reef Studies, Crawley, WA, Australia; 2 School of Earth Sciences, The Ohio State University, Columbus, Ohio, United States of America; 3 School of Marine Science and Policy, University of Delaware, Lewes, Delaware, United States of America; King Abdullah University of Science and Technology, Saudi Arabia

## Abstract

Coral skeletal boron isotopes have been established as a proxy for seawater pH, yet it remains unclear if and how this proxy is affected by seawater temperature. Specifically, it has never been directly tested whether coral bleaching caused by high water temperatures influences coral boron isotopes. Here we report the results from a controlled bleaching experiment conducted on the Caribbean corals *Porites divaricata, Porites astreoides*, and *Orbicella faveolata*. Stable boron (δ^11^B), carbon (δ^13^C), oxygen (δ^18^O) isotopes, Sr/Ca, Mg/Ca, U/Ca, and Ba/Ca ratios, as well as chlorophyll *a* concentrations and calcification rates were measured on coral skeletal material corresponding to the period during and immediately after the elevated temperature treatment and again after 6 weeks of recovery on the reef. We show that under these conditions, coral bleaching did not affect the boron isotopic signature in any coral species tested, despite significant changes in coral physiology. This contradicts published findings from coral cores, where significant decreases in boron isotopes were interpreted as corresponding to times of known mass bleaching events. In contrast, δ^13^C and δ^18^O exhibited major enrichment corresponding to decreases in calcification rates associated with bleaching. Sr/Ca of bleached corals did not consistently record the 1.2°C difference in seawater temperature during the bleaching treatment, or alternatively show a consistent increase due to impaired photosynthesis and calcification. Mg/Ca, U/Ca, and Ba/Ca were affected by coral bleaching in some of the coral species, but the observed patterns could not be satisfactorily explained by temperature dependence or changes in coral physiology. This demonstrates that coral boron isotopes do not record short-term bleaching events, and therefore cannot be used as a proxy for past bleaching events. The robustness of coral boron isotopes to changes in coral physiology, however, suggests that reconstruction of seawater pH using boron isotopes should be uncompromised by short-term bleaching events.

## Introduction

The world's oceans are simultaneously warming and acidifying at unprecedented pace due to rising atmospheric CO_2_ concentrations [1], thereby severely threatening marine ecosystems [2], [3]. Coral reefs are particularly vulnerable because they are highly sensitive to changes in both seawater temperature and pH [4]–[6].

Coral reefs are increasingly subject to periods with unusually warm seawater temperatures which can cause coral bleaching, defined as a significant loss of photosynthetic pigments and/or algal endosymbionts from the coral tissue [7], . Although corals can sometimes recover from bleaching events [13]–[18], other corals are significantly impacted by bleaching events, resulting in widespread mortality for more sensitive species [10], [11], [19]. As surface ocean temperatures have already increased by 0.6°C since preindustrial times and are projected to increase by at least another 2.0°C under a business as usual scenario by the year 2100 [1], coral bleaching events are expected to increase in frequency and intensity [11], [20]–[22], thus contributing to the worldwide decline of corals reefs [23], [24].

In addition to greenhouse-induced warming, rising levels of atmospheric CO_2_ concentrations have already caused a drop in surface seawater pH by approximately 0.1 unit compared to preindustrial times [25], and a further decrease of 0.3 pH units by the end of this century has been predicted [1], [26]. This is of particular concern for marine calcifying organisms including corals because ocean acidification (OA) typically compromises calcification [27]–[33]. Although it is now recognised that scleractinian corals have a capacity to internally up-regulate pH [34]–[40], it has been estimated that coral calcification will decrease by up to 22% by the year 2100 [41]. Therefore, predicting how global climate change will affect ocean chemistry and marine ecosystems in the coming decades is of imminent concern and requires understanding of how factors such as ocean temperature and pH have varied naturally in the past. Since direct measurements of ocean chemistry are only available for the most recent decades, proxy records are of crucial importance.

Coral skeletal boron isotopes (δ^11^B) are promising proxies of seawater pH [31], [34], [40], [42]–[45] that have been used to reconstruct paleo-pH [46]–[48]. Their use as a pH-proxy is based on the fact that the relative abundance of the two major aqueous boron species in seawater, boric acid (B(OH)_3_) and borate ion (B(OH)_4_^−^), are dependent on seawater pH. Further, their isotopic composition is also pH dependent and differs by about 27‰. It is generally thought that corals mainly incorporate the charged borate ion into the skeleton as their isotopic composition reflects that of borate [34],[42],[49], although this has been questioned by some studies [50], [51]. However, it is now established that coral skeletal δ^11^B does not directly record seawater pH but rather reflects the pH at the site of calcification [34], [35], [40], [42], which is typically elevated by up to 1 unit [35], [37]–[39] or more [36] relative to ambient seawater. Therefore, species-specific calibrations have to be performed to quantify the amount of pH-upregulation in order to reconstruct seawater pH [34].

One key requirement for any proxy is that it faithfully records the environmental variable of interest without being influenced by other factors. Several experimental studies have attempted to identify potentially confounding effects of light, depth, temperature, and feeding on coral δ^11^B but, taken together, their results are contradictory. For example, Hönisch et al. [45] showed that various light, depth, and feeding regimes did not influence δ^11^B of *Porites compressa* and *Montipora compressa*. However, another study using branching *Acropora* sp. found that δ^11^B decreased as light intensity increased, introducing a bias of about 0.05 pH units [43].

Regarding temperature, Reynaud et al. [44] observed no temperature effect on δ^11^B of *Acropora* sp. cultured at 25 and 28°C and two pCO_2_ levels. Interestingly, Dissard et al. [43] cultured *Acropora* sp. using the same protocol as in Reynaud et al. [44] at 22, 25 and 28°C, and found a significant temperature effect only at the lower temperatures of 22 and 25°C, corresponding to an increase in δ^11^B of about 0.02 pH units. A significant temperature effect was not observed between 25 and 28°C, consistent with the findings of Reynaud et al. [44]. It therefore appears that other factors may have confounded this work or, at least in *Acropora*, that temperature effects on coral δ^11^B are non-linear and only exist for lower temperature ranges. However, given that both studies only used nubbins prepared from a single parent colony of one species, it is not clear whether these results could be reproduced if nubbins were used from multiple parent colonies (thus representing genetically different individuals), and/or different species of coral such as massive *Porites* sp., which is frequently used for paleoclimate reconstruction.

Although the experimental evidence available to date suggests that temperature may only affect coral δ^11^B at lower temperatures (i.e., <25°C), no studies have examined stressfully high temperatures that can result in coral bleaching. Evidence from a massive *Porites* core from the Great Barrier Reef suggests that significant short-term decreases in δ^11^B could be the result of coral bleaching events [47]. They proposed that this was due to a major reduction in pH at the site of calcification associated with impaired calcification, and suggest that coral δ^11^B may be used as a proxy for past bleaching events [47]. Although coral δ^13^C and δ^18^O as well as Sr/Ca have been suggested as potential bleaching proxies, they were unreliable in this regard or in the case of Sr/Ca lack sufficient detail [52]–[55]. Therefore, establishing coral δ^11^B as a bleaching proxy could present a major step forward in understanding how often bleaching events occurred in the past. Further, it is essential that temperature effects on coral δ^11^B be better understood over a large range of temperatures and using a variety of coral species to improve the reconstruction of past seawater pH.

To address these questions, we conducted a controlled, replicated bleaching experiment using the Caribbean coral species *Porites divaricata*, *Porites astreoides* and *Orbicella faveolata*. Coral skeletal δ^11^B, δ^13^C and δ^18^O as well as Sr/Ca, Mg/Ca, U/Ca, and Ba/Ca were analysed in samples collected immediately after the bleaching experiment and again after 6 weeks of recovery on the reef. Several physiological measurements were concurrently performed on these corals [13], [56]–[59], providing a rich background of physiological information within which to interpret our findings. Based on the evidence presented by Wei et al. [47], we hypothesized that δ^11^B of bleached corals decreases compared to non-bleached controls, and approaches (i.e., increases to) the values of non-bleached controls during recovery.

## Methods

### Coral Bleaching Experiment

The corals used for this study were taken from an experiment where three species of Caribbean corals were experimentally bleached in two consecutive summers [13]. These corals were physiologically fully recovered (i.e., there were no significant differences between treatment and control corals in any of the measured variables) after a year on the reef prior to exposure to the elevated temperature stress for a second time [57], [59]. A detailed description of the experimental design can be found in Grottoli et al. [13].

Briefly, coral fragments were collected from 9 healthy colonies of *Porites divaricata* (branching morphology), *Porites astreoides* (mounding/encrusting morphology), and mounding *Orbicella faveolata* (formerly *Montastraea faveolata*) [60] (large mounding morphology) in July 2009 from reefs near Puerto Morelos, Yucatan Peninsula, Mexico (20°50′N, 86°52′W). All collections and experiments were conducted following the rules and regulations of Mexico and imported to the USA under CITES permits held by UNAM-ICML and the Ohio State University. Half of the coral fragments from each parent colony were randomly assigned to each treatment: (1) ambient control fragments were maintained in tanks with ambient seawater temperature (30.66 ± 0.24°C), and (2) treatment fragments were placed in tanks with elevated seawater temperature (31.48 ± 0.20°C). Seawater temperature in the treatment tanks was gradually elevated over the course of a week. Corals were not fed but had access to unfiltered seawater. High-precision seawater pH measurements were not made. After a total of 15 days, temperature in all tanks was returned to ambient levels, and all coral fragments were placed back *in situ* on the reef for one year.

In July 2010, the experiment was repeated. All corals that had served as ambient control fragments the previous summer were placed in tanks with ambient seawater (30.40 ± 0.23°C), whereas all corals that had been used as treatment fragments were maintained in tanks with elevated temperature (31.60 ± 0.24°C). After 17 days, all tanks were returned to ambient temperature levels and one control and one treatment fragment per colony of each species were then frozen for geochemical analyses ( = 0 weeks on the reef). All remaining fragments were placed on the back reef. All remaining corals (i.e., one control and one treatment fragment per colony of each species) were recollected from the reef after 6 weeks and frozen for geochemical analyses.

### Physiological Analyses

#### Chlorophyll *a*

Coral tissue was removed from the skeleton of a portion of each fragment with a WaterPik, homogenized, and centrifuged to separate the algal endosymbiont from the animal tissue. A subsample of the resuspended algal pellet was broken using glass beads in 100% acetone and subsequently stored at −20°C overnight. Chlorophyll *a* was then determined using a Shimadzu UV-VIS spectrophotometer and the equations of Jeffrey and Humphrey [61]. Chlorophyll *a* content was standardized to surface area, which was determined using the aluminium foil method [62].

#### Calcification

Published calcification rates determined using the buoyant weight technique [63] were reproduced from Grottoli et al. [13] with permission of the publisher (license 3424840151859).

### Isotopic Analyses

Coral tissue was removed from the skeleton using a dental hygiene tool. The uppermost layer (approx. 0.25–0.5 mm) of the dried skeleton was then gently shaved with a diamond-tipped Dremel tool and ground to fine powder using agate mortar and pestles [52], [64]. Approximately 2–11 mg of the skeletal powder was weighed in pre-cleaned, pre-weighed Teflon tubes. An aliquot of this powder was used for δ^13^C and δ^18^O analyses (see below). When there was <2 mg of powder available from individual fragments, samples from multiple fragments of the same species and time point were combined for δ^11^B analyses.

#### Boron isotopes

The following procedures were carried out in a clean lab. To remove organic matter, samples were rinsed with H_2_O, incubated with 2 ml of 6.25% NaClO for 15 min including 5 min of sonication, then centrifuged for 2 min at highest speed, and the supernatant removed. Powders were then rinsed 3× with H_2_O, with each rinse including 5 min sonication and 2 min centrifugation at highest speed. After cleaning, tubes were lightly capped and samples dried to constant dry weight by placing them on a 60°C hotplate with a heat-lamp next to it. Cleaned samples were dissolved in 0.58 N HNO_3_. An aliquot of this solution was then diluted for trace element measurements (see below), and the remaining solution extracted for boron using ion exchange resins [35], [65].

The extracted boron was analysed at the University of Western Australia using either a Neptune Plus Multi-Collector Inductively Coupled Plasma Mass Spectrometer (MC-ICP-MS; Thermo Fisher Scientific) fitted with a PFA nebulizer and a cyclonic quartz spray chamber or a NU Plasma II MC-ICP-MS (NU Instruments). The boron isotopic composition of the skeleton (δ^11^B) was reported as the per mil deviation of the stable isotopes ^11^B:^10^B relative to SRM-951. Sample measurements were bracketed by SRM-951 enriched to 24.7‰ and blank measurements [66], and all samples were analysed in duplicate. Repeated measurements of SRM-951 had a standard deviation (1σ) of ± 0.37‰ (n = 124) for concentrations ranging from 100–200 ppb B.

#### Carbon and oxygen isotopes

A 80–100 µg aliquot of the dried and ground skeletal powder was analysed for δ^13^C and δ^18^O using an automated Kiel Carbonate Device coupled to a Stable Isotope Ratio Mass Spectrometer (SIRMS; Finnigan Delta IV) at The Ohio State University. Samples were not pre-treated [67]. Samples were acidified under vacuum with 100% ortho-phosphoric acid. The carbon isotopic composition of the skeleton (δ^13^C) was reported as the per mil deviation of the stable isotopes ^13^C:^12^C relative to Vienna-Peedee Belemnite Limestone standard (v-PDB). Skeletal oxygen isotopes (δ^18^O) were reported as the per mil deviation of the stable isotopes ^18^O:^16^O relative to v-PDB. Approximately 10% of all samples were run in duplicate. The standard deviation (1σ) of repeated measurements of internal standards was ± 0.03‰ for δ^13^C and ± 0.07‰ for δ^18^O (n = 55).

### Trace Element Analyses

From the solution used for δ^11^B analysis, a 2–7 µL aliquot was diluted to a final concentration of 10 ppm Ca in 2% HNO_3_ spiked with ∼19 ppb Sc, 19 ppb Y, 0.19 ppb Pr, 0.095 ppb Bi, and 19 ppb V. Samples were then analysed for Sr/Ca, Mg/Ca, U/Ca, and Ba/Ca on an X-Series 2 Quadrupole Inductively Coupled Plasma Mass Spectrometer (Q-ICPMS; Thermo Fisher Scientific) at the University of Western Australia using the standard Xt interface and the plasma screen fitted. Between-run reproducibility (RSD) of a coral standard subjected to the same chemistry as the coral samples was 0.4, 0.4, 0.8 and 0.4% for Sr/Ca, Mg/Ca, U/Ca and Ba/Ca, respectively (n = 6).

### Statistical Analyses

Two-way analysis of variance (ANOVA) was used to test for the effects of temperature and time on coral chlorophyll *a*, δ^11^B, δ^13^C, δ^18^O, Sr/Ca, Mg/Ca, U/Ca, and Ba/Ca. Temperature was fixed and fully crossed with two levels (treatment, control), and time was fixed and fully crossed with two levels (0, 6 weeks on the reef). Residual values for each variable and species were calculated and tested for normality using a Shapiro-Wilk's test and homogeneity of variance was assessed with plots of expected versus residual values. Non-normal data sets were transformed. Post hoc Tukey tests were performed when main effects – but no interaction terms - were significant. A posteriori slice tests (e.g., tests of simple effects) [68] determined if the treatment and control averages significantly differed at each time interval. Calcification data was previously analysed in Grottoli et al. [13].

Corals were considered to be fully recovered once average chlorophyll *a*, δ^11^B, δ^13^C, δ^18^O, Sr/Ca, Mg/Ca, U/Ca, and Ba/Ca values of treatment corals no longer significantly differed from the average values of controls. Since all fragments were exposed to identical conditions except temperature during the tank portion of the experiment, any differences in the observed responses were due to temperature effects alone and independent of seasonal variation. Bonferroni corrections were not applied because they increase the risk of false negatives [69], [70]. *P*-values ≤0.05 were considered significant. Statistical analyses were performed using SAS software, version 9.3 of the SAS System for Windows.

## Results

### Physiology

**Figure 1:**
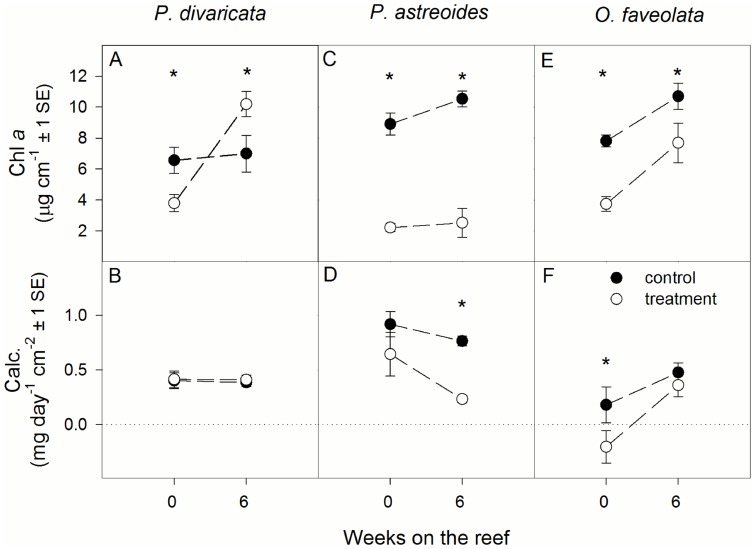
Average area-normalized chlorophyll a ( = chl a) concentration and calcification ( = calc.) rate of (A, B) Porites divaricata, (C, D) Porites astreoides, and (E, F) Orbicella faveolata after 0 and 6 weeks on the reef. Asterisks indicate significant differences between control and treatment corals at a specific time interval. Sample size ranges from 6–9. Calcification data reproduced from Grottoli et al. [13] with permission of the publisher.

A significant interaction of temperature and time was observed for area-normalized chlorophyll *a* concentrations of *P. divaricata*, with treatment corals having 42% lower and 46% higher chlorophyll *a* concentrations than controls after 0 and 6 weeks on the reef, respectively (Fig. 1A, Table 1). Calcification rates were the same for both treatment and control *P. divaricata* throughout the study (Fig. 1B) [13].

**Table 1:** Results of two-way ANOVA for area-normalized chlorophyll *a* content of *P. divaricata, P. astreoides*, and *O. faveolata*.

Variable	Effect	df	SS	*F*-statistic	*p*-value	Tukey
***P. divaricata***						
Chlorophyll *a*	Model	3, 28	164.4113	9.82	**0.0002**	
	Temp.	1	0.3307	0.06	0.8097	
	Time	1	82.8258	14.84	**0.0007**	
	Temp. x Time	1	63.1744	11.32	**0.0025**	
***P. astreoides***						
Chlorophyll *a*	Model	3, 33	469.1181	43.44	**<0.0001**	
	Temp.	1	456.8590	126.91	**<0.0001**	CO>TR
	Time	1	7.9244	2.20	0.1483	
	Temp. x Time	1	3.6588	1.02	0.3214	
***O. faveolata***						
Chlorophyll *a*	Model	3, 33	206.4635	12.44	**<0.0001**	
	Temp.	1	106.3292	19.22	**0.0001**	CO>TR
	Time	1	99.1921	17.93	**0.0002**	6>0
	Temp. x Time	1	2.4176	0.44	0.5136	

The effect of temperature (Temp.) was fixed with two levels (CO  =  control 30.4°C, TR  =  treatment 31.6°C), and time was fixed with 2 levels (0, 6 weeks). When main effects (but no interaction terms) were significant, Tukey post hoc results are shown. Significant *p*-values (*p*≤0.05) are highlighted in bold. df  =  degrees of freedom, SS  =  sum of squares of the effect.

In *P. astreoides*, area-normalized chlorophyll *a* concentrations were influenced by temperature, with treatment corals having 75% and 76% lower chlorophyll *a* rates than the controls at both 0 and 6 weeks on the reef, respectively (Fig. 1C, Table 1). Calcification rates of treatment corals were the same as in controls initially, but were 69% lower after 6 weeks on the reef (Fig. 1D) [13].

In *O. faveolata*, area-normalized chlorophyll *a* concentrations were influenced by both temperature and time (Table 1). They were 52% and 28% lower in treatment corals compared to controls at 0 and 6 weeks on the reef, respectively (Fig. 1E, Table 1). Further, they were higher at 6 weeks on the reef compared to 0 weeks (Fig. 1E, Table 1). Calcification rates of treatment corals were significantly lower (−212%) than in controls initially but had fully recovered 6 weeks later (Fig. 1F) [13].

### Isotopes

**Figure 2:**
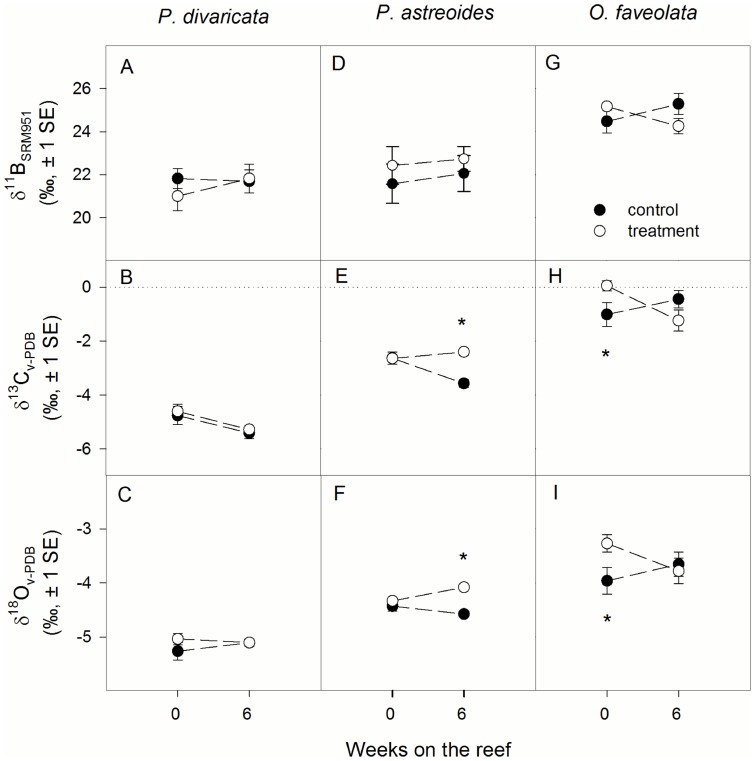
Average δ^11^B, δ^13^C, and δ^18^O of (A–C) Porites divaricata, (D–F) Porites astreoides, and (G–I) Orbicella faveolata after 0 and 6 weeks on the reef. Asterisks indicate significant differences between control and treatment corals at a specific time interval. Sample size ranges from 3–9.

δ^11^B was not influenced by temperature or time in any of the three species (Table 2, Fig. 2A, D, G). Similarly, δ^13^C and δ^18^O of *P. divaricata* were not affected by either temperature or time (Table 2, Fig. 2B, C). However, a significant interaction of temperature and time was observed for δ^13^C and δ^18^O of *P. astreoides* (Table 2, Fig. 2E, F). Initially, δ^13^C and δ^18^O did not differ between treatment and control corals, but after 6 weeks on the reef, both δ^13^C and δ^18^O were significantly higher in treatment compared to control corals (Fig. 2E, F). In *O. faveolata*, δ^13^C and δ^18^O were not influenced by temperature or time, but post hoc slice tests revealed that treatment corals had higher values than the controls at 0 weeks on the reef (Fig. 2H, I, Table 2).

**Table 2:** Results of two-way ANOVAs for δ^11^B, δ^13^C, and δ^18^O of *P. divaricata, P. astreoides*, and *O. faveolata*.

Variable	Effect	df	SS	*F*-statistic	*p*-value
***P. divaricata***					
δ^11^B	Model	3, 19	2.7948	0.46	0.7148
	Temp.	1	0.5602	0.28	0.6066
	Time	1	0.5602	0.28	0.6066
	Temp. x Time	1	1.0643	0.52	0.4795
δ^13^C	Model	3, 23	2.8226	2.76	0.0689
	Temp.	1	0.1219	0.36	0.5565
	Time	1	2.5603	7.51	**0.0126**
	Temp. x Time	1	0.0008	0.00	0.9610
δ^18^O	Model	3, 23	0.1570	0.89	0.4619
	Temp.	1	0.0778	1.33	0.2628
	Time	1	0.0111	0.19	0.6683
	Temp. x Time	1	0.0778	1.33	0.2628
***P. astreoides***					
δ^11^B	Model	3, 22	3.8106	0.32	0.8094
	Temp.	1	3.2908	0.83	0.3725
	Time	1	0.8574	0.22	0.6464
	Temp. x Time	1	0.0420	0.01	0.9189
δ^13^C	Model	3, 34	7.2196	10.56	**<0.0001**
	Temp.	1	2.9506	12.95	**0.0011**
	Time	1	1.0442	4.58	**0.0403**
	Temp. x Time	1	3.0344	13.32	**0.0010**
δ^18^O	Model	3, 34	1.1779	10.85	**<0.0001**
	Temp.	1	0.7728	21.36	**<0.0001**
	Time	1	0.0240	0.66	0.4217
	Temp. x Time	1	0.3460	9.56	**0.0042**
***O. faveolata***					
δ^11^B	Model	3, 13	2.6525	1.25	0.3430
	Temp.	1	0.0981	0.14	0.7173
	Time	1	0.0086	0.01	0.9145
	Temp. x Time	1	2.5358	3.59	0.0876
δ^13^C	Model	3, 33	8.3114	2.56	0.0739
	Temp.	1	0.1528	0.14	0.7100
	Time	1	1.0742	0.99	0.3275
	Temp. x Time	1	7.3876	6.82	**0.0140**
δ^18^O	Model	3, 33	2.1365	1.69	0.1903
	Temp.	1	0.6800	1.61	0.2138
	Time	1	0.0845	0.20	0.6576
	Temp. x Time	1	1.4076	3.34	0.0776

The effect of temperature (Temp.) was fixed with two levels (control 30.4°C, treatment 31.6°C), and time was fixed with 2 levels (0, 6 weeks). Significant *p*-values (*p*≤0.05) are highlighted in bold. df  =  degrees of freedom, SS  =  sum of squares of the effect.

### Trace Elements

**Figure 3:**
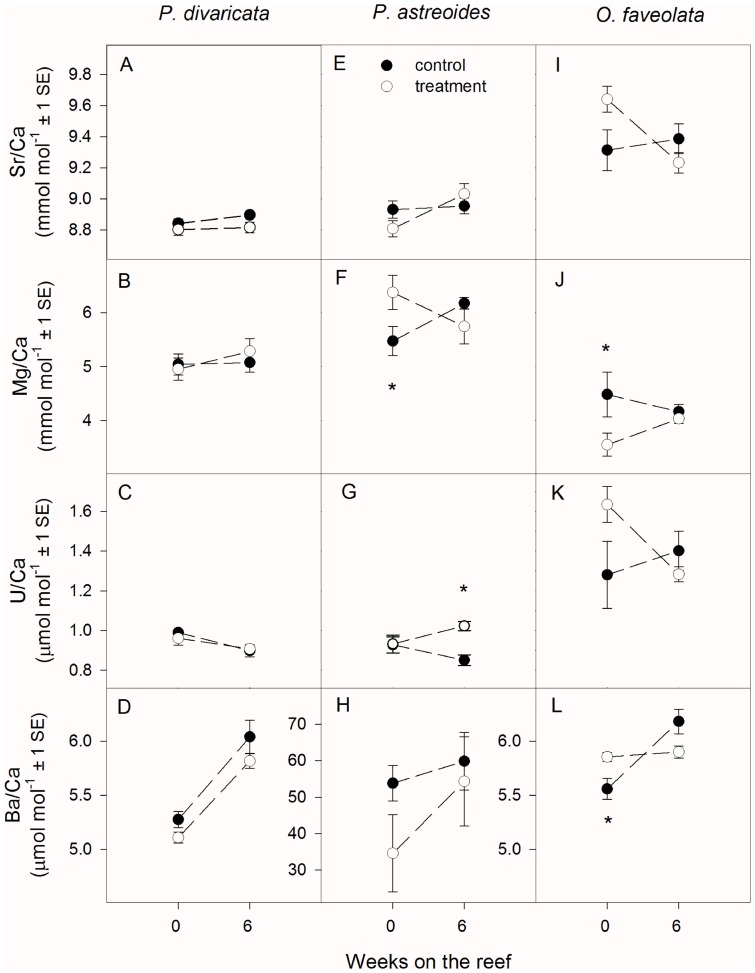
Average Sr/Ca, Mg/Ca, U/Ca, and Ba/Ca of (A–D) Porites divaricata, (E–H) Porites astreoides, and (I–L) Orbicella faveolata after 0 and 6 weeks on the reef. Asterisks indicate significant differences between control and treatment corals at a specific time interval. Sample size ranges from 3–7. Note that the Y-axis for Ba/Ca differs for P. astreoides and the other two species.

In *P. divaricata*, Sr/Ca, Mg/Ca, and U/Ca were not affected by either temperature or time (Table 3, Fig. 3A-C). However, Ba/Ca was affected by both temperature and time, with higher ratios found in controls than in treatment corals and increases in Ba/Ca after 6 weeks on the reef compared to 0 weeks (Table 3, Fig. 3D).

**Table 3:** Results of two-way ANOVAs for Mg/Ca, Sr/Ca, Ba/Ca, and U/Ca of *P. divaricata, P. astreoides,* and *O. faveolata*.

Variable	Effect	df	SS	*F*-statistic	*p*-value	Tukey
***P. divaricata***						
Sr/Ca	Model	3, 19	0.0241	1.49	0.2557	
	Temp.	1	0.0170	3.15	0.0950	
	Time	1	0.0055	1.02	0.3273	
	Temp. x Time	1	0.0019	0.35	0.5622	
Mg/Ca	Model	3, 19	0.2820	0.42	0.7427	
	Temp.	1	0.0170	0.08	0.7868	
	Time	1	0.1575	0.70	0.4152	
	Temp. x Time	1	0.1045	0.46	0.5054	
U/Ca	Model	3, 19	0.0264	2.02	0.1520	
	Temp.	1	0.0005	0.10	0.7504	
	Time	1	0.0252	5.78	**0.0287**	
	Temp. x Time	1	0.0017	0.39	0.5435	
Ba/Ca	Model	3, 19	2.8756	27.77	**<0.0001**	
	Temp.	1	0.1797	5.21	**0.0365**	CO>TR
	Time	1	2.5673	74.38	**<0.0001**	6>0
	Temp. x Time	1	0.0038	0.11	0.7456	
***P. astreoides***						
Sr/Ca	Model	3, 22	0.1453	2.69	0.0751	
	Temp.	1	0.0029	0.16	0.6926	
	Time	1	0.0860	4.78	**0.0415**	
	Temp. x Time	1	0.0570	3.17	0.0911	
Mg/Ca	Model	3, 22	2.7689	2.47	0.0929	
	Temp.	1	0.3173	0.85	0.3681	
	Time	1	0.0069	0.02	0.8934	
	Temp. x Time	1	2.5033	6.71	**0.0180**	
U/Ca	Model	3, 22	0.0872	4.11	**0.0210**	
	Temp.	1	0.0442	6.24	**0.0218**	
	Time	1	0.0003	0.04	0.8529	
	Temp. x Time	1	0.0398	5.61	**0.0286**	
Ba/Ca	Model	3, 21	2242.8826	1.63	0.2181	
	Temp.	1	808.5285	1.76	0.2012	
	Time	1	871.9859	1.90	0.1851	
	Temp. x Time	1	246.0976	0.54	0.4736	
***O. faveolata***						
Sr/Ca	Model	3, 13	0.2863	2.48	0.1206	
	Temp.	1	0.0259	0.67	0.4308	
	Time	1	0.0962	2.50	0.1447	
	Temp. x Time	1	0.1983	5.16	**0.0464**	
Mg/Ca	Model	3, 13	1.5265	1.94	0.1879	
	Temp.	1	0.9719	3.70	0.0835	
	Time	1	0.0238	0.09	0.7696	
	Temp. x Time	1	0.5522	2.10	0.1779	
U/Ca	Model	3, 13	0.2635	1.69	0.2309	
	Temp.	1	0.0479	0.92	0.3591	
	Time	1	0.0458	0.88	0.3696	
	Temp. x Time	1	0.1922	3.71	0.0831	
Ba/Ca	Model	3, 13	0.7797	8.77	**0.0038**	
	Temp.	1	0.0001	0.00	0.9463	
	Time	1	0.3844	12.97	**0.0048**	
	Temp. x Time	1	0.2847	9.61	**0.0113**	

The effect of temperature (Temp.) was fixed with two levels (CO  =  control 30.4°C, TR  =  treatment 31.6°C), and time was fixed with 2 levels (0, 6 weeks). When main effects (but no interaction terms) were significant, Tukey post hoc results are shown. Significant *p*-values (*p*≤0.05) are highlighted in bold. df  =  degrees of freedom, SS  =  sum of squares of the effect.

In *P. astreoides*, Sr/Ca, Mg/Ca and Ba/Ca were not affected by either temperature or time (Table 3, Fig. 3E, F, H). However, post hoc slice tests revealed that Mg/Ca ratios were higher (+16.5%) in treatment than in control corals at 0 weeks (Table 3, Fig. 3F). A significant interaction between temperature and time was observed for U/Ca, with ratios being significantly higher (+20.3%) in treatment than in control corals after 6 weeks on the reef (Table 3, Fig. 3G).

In *O. faveolata*, Sr/Ca, Mg/Ca and U/Ca were not influenced by either temperature or time (Table 3, Fig. 3I-K). However, post hoc slice tests revealed that treatment Mg/Ca ratios were lower (−26.3%) than the controls at 0 weeks on the reef (Fig. 3J). A significant interaction of temperature and time was observed for Ba/Ca, with treatment values being higher (+5.8%) than the control initially, but no longer different after 6 weeks on the reef (Fig. 3L, Table 3).

## Discussion

### Isotopes

Coral skeletal boron isotopes have been established as a proxy for seawater pH, yet it remains unclear if and how this proxy is affected by seawater temperature. Specifically, it has never been tested whether coral bleaching due to elevated temperature affects coral δ^11^B. Here, we show for the first time that experimental coral bleaching does not affect δ^11^B of three Caribbean coral species (Fig. 2A, D, G). This finding does not support our initial hypothesis based on evidence presented by Wei et al. [47] that coral δ^11^B may decrease significantly during coral bleaching events. However, it is in agreement with experimental studies showing that δ^11^B is not affected by moderate increases in temperatures from 25°C to 28°C [43], [44], which do not induce coral bleaching.

Coral bleaching is caused by a significant loss of algal endosymbionts and/or photosynthetic pigments from the coral tissue [7], . In the current study, treatment corals of all species had significantly lower area-normalized chlorophyll *a* levels than controls throughout the study (Fig. 1A, C, E), primarily driven by lower endosymbiont densities [13]. Further, treatment corals of all species had a significantly lower maximum quantum yield of photosystem II (F_v_/F_m_) than controls for up to 6 weeks on the reef [59]. Thus, treatment corals of all three species were bleached. This demonstrates that even though corals experienced significant temperature stress and bleaching, coral skeletal δ^11^B remained unaffected.

Calcification rates are often compromised in bleached corals [17], [52], [54], [71], and may therefore have affected the response of skeletal isotopes and trace elements [72], [73]. Indeed, calcification rates of treatment *O. faveolata* were significantly lower than in the controls at 0 weeks on the reef (Fig. 1D). In *P. astreoides*, calcification was not significantly lower than the controls until after 6 weeks on the reef (Fig. 1F) because the bleaching response of the animal host showed a lag of several weeks in this coral species [13]. Such lags in the bleaching response have been previously observed in three species of Hawaiian corals as well [17], [52]. However, none of these declines in calcification resulted in significant differences in δ^11^B between treatment and control corals (Fig. 2D, G) despite the fact that treatment corals were significantly bleached at this time (Fig. 1C, E). Similarly, when calcification rates were maintained in bleached corals, as was the case in *P. divaricata* and in *P. astreoides* initially at the end of the heating period (Fig. 1B, D), δ^11^B of treatment corals did not differ from the controls (Fig. 2A, D). Based on these results, coral δ^11^B is unaffected by calcification rate.

The insensitivity of δ^11^B to calcification rates is further corroborated by the fact that both skeletal δ^13^C and δ^18^O changed in response to decreases in calcification rates in two species, yet δ^11^B did not (Figs. 1, 2). In treatment *P. astreoides* and *O. faveolata*, both δ^13^C and δ^18^O increased as calcification decreased. This is unexpected given that both δ^13^C and δ^18^O are assumed to decrease when photosynthesis decreases and seawater temperature increases (provided there were no changes in salinity), respectively [74]–[79]. Instead, the enrichment in both δ^13^C and δ^18^O indicates that skeletal carbonate started approaching isotopic equilibrium with seawater as calcification rates slowed down [76] and that calcification rate was the primary control on δ^13^C and δ^18^O. Similar relationships between skeletal δ^13^C, δ^18^O, and calcification were also noted in other corals when bleached [52], [80]. For *O. faveolata*, this strongly suggests that some skeletal material was deposited during the bleaching treatment, even though on average net calcification rates were slightly negative. Further, in *P. divaricata*, δ^13^C and δ^18^O were unaffected by bleaching at any point, which is consistent with no changes in calcification rates.

Clearly, the carbon and oxygen isotopic signal was dominated by kinetic effects associated with calcification rates [76] in all three species. Kinetic effects likely overpowered any metabolic effects in δ^13^C due to reduced chlorophyll *a* concentrations (implying reduced photosynthesis rates) as well as the expected temperature-dependent effect on δ^18^O [74]. Although a simple data correction has been proposed to correct for kinetic isotope effects in δ^13^C [81], it does not reliably remove kinetic isotope effects in bleached corals [82]. Unfortunately, these findings confirm that coral δ^13^C and δ^18^O cannot be used as a proxy for past bleaching events [17], [52], [54], [55], and that coral δ^11^B does not record bleaching events as suggested by Wei et al. [47], at least not in the coral species studied here.

The observed lack of a bleaching effect on coral δ^11^B suggests that coral bleaching – and therefore the physiological status of the algal endosymbiont - does not affect pH-upregulation at the site of calcification. This is surprising given that coral calcification is enhanced in the light via photosynthesis [83]–[85] due to a variety of mechanisms including removal of protons from the site of calcification, supply of ATP for ion transport and/or organic matrix synthesis, production of oxygen, supply of precursors for organic matrix production, and removal of phosphates [86], [87]. Thus, if the symbiotic relationship is disrupted during bleaching, any of the mechanisms listed above would likely be interrupted as well, including the impaired removal of protons from the site of calcification that affects δ^11^B values. Clearly, this was not the case in the coral species studied here, which further suggests that decreased calcification rates of bleached corals may not be due to impaired control over the calcifying environment.

Heterotrophy and energy reserves can promote many physiological aspects including calcification in healthy corals [88], and also enhances resistance to and recovery from coral bleaching [13], [18], [89], [90]. It is therefore possible that heterotrophic energy input may have helped bleached corals to maintain pH-upregulation at the site of calcification. However, feeding rates and total carbon acquired by feeding relative to metabolic demand (CHAR) did not differ between treatment and control corals immediately after the bleaching treatment in any of the species [13]. It is possible that other heterotrophic sources of carbon such as dissolved and particulate organic carbon (DOC and POC, respectively) could be providing the necessary supplemental energy for calcification in bleached corals. For example, Levas [57] showed that DOC can provide 15% of daily metabolic carbon in bleached *O. faveolata* corals.

Collectively, these results demonstrate that pH-upregulation at the site of calcification is robust to significant physiological changes caused by short bleaching events. This suggests either that this process has high priority during energy allocation even under scenarios of resource limitation such as coral bleaching, or that pH-upregulation does not require that much energy and can thus be maintained during coral bleaching. However, it is possible that when coral bleaching events are prolonged, a certain threshold of resource limitation may be reached at which corals are no longer able to maintain pH-upregulation at the site of calcification. Resource limitation in this scenario would not only be related to a decrease in transferred carbon by the algal endosymbiont [18], [91], but also to oxygen limitation [92], [93] and potentially other mechanisms involved in light-enhanced calcification that would be interrupted due to bleaching. This would then result in decreased δ^11^B values of bleached corals, consistent with observations by Wei et al. [47] for a *Porites* coral core, where significant decreases in boron isotopes were interpreted as corresponding to the 1998 mass bleaching event on the Great Barrier Reef. This threshold, if existent, would likely be on the order of at least several months because the physiology of *P. astreoides* in this study was affected for at least 6 weeks [13], yet showed no decrease in boron isotopes. Further, several studies have shown that coral growth rates can take several years to fully recover from bleaching [71], [80], [94], [95].

### Trace Elements

Coral bleaching did not affect trace element composition (Sr/Ca, Mg/Ca, U/Ca, and Ba/Ca) of *P. divaricata*, but influenced Mg/Ca and U/Ca ratios of *P. astreoides* as well as Mg/Ca and Ba/Ca in *O. faveolata* (Fig. 3). The lack of any temperature effect on Sr/Ca is particularly surprising, because it is generally considered to be one of the most reliable sea surface temperature (SST) proxies available [96], [97], [98], although it is not free of complications [99]–[105]. Sr/Ca sensitivity is about 0.04–0.08 mmol mol^−1^ per °C [100], [101], [106]–[109]. Thus the treatment corals in this study were expected to have Sr/Ca ratios that were 0.05–0.1 mmol mol^−1^ lower than the control corals given the inverse correlation between temperature and Sr/Ca. At 0 weeks on the reef, treatment *P. divaricata* and *P. astreoides* had indeed lower Sr/Ca ratios than the controls (0.04 and 0.12 mmol mol^−1^, respectively), whereas treatment *O. faveolata* had higher Sr/Ca values (0.33 mmol mol^−1^). However, none of these differences were statistically significant (Fig. 3A, E, I).

During cold and hot temperature stress, sharp increases in Sr/Ca have been observed in *Porites* coral from the Great Barrier Reef [53], [96]. Marshall and McCulloch [53] proposed that temperature stress disrupts the Sr/Ca – SST relationship by inhibiting Ca transport enzymes, whereas Sr transport is unaffected. Thus, the Sr/Ca ratio increases because relatively less Ca is made available to the calcifying fluid. Similarly, Cohen et al. [99] suggested that endosymbiont photosynthesis enhances Ca but not Sr transport to the calcifying fluid, thus resulting in lower Sr/Ca ratios during times of high photosynthesis. Following this reasoning, Sr/Ca should therefore increase in bleached corals when photosynthesis is impaired and calcification slows down. However, the findings from this study do not generally support this hypothesis. Even though Sr/Ca ratios were higher in treatment than in control *O. faveolata* initially (Fig. 3I) when chlorophyll *a* concentrations and calcification rates were significantly lower (Fig. 1F), this difference was not statistically significant (*p* = 0.0536). Marshall and McCulloch [53] suggested that a particular temperature threshold needs to be reached before Sr/Ca ratios are affected by temperature stress. Potentially, this threshold differs for different coral species and therefore resulted in equivocal results here.

Changes in calcification rates due to coral bleaching may have influenced Sr/Ca [100], [101], [110]. Slower growing corals typically have higher Sr/Ca values than faster growing corals [100], [101], [110]. However, this was not generally observed, and many other studies also failed to detect growth rate effects on Sr/Ca [102], [106], [111]–[113]. It therefore seems that overall coral skeletal Sr/Ca is not influenced by the physiological changes or temperature changes occurring during short coral bleaching events.

Mg/Ca ratios were affected by coral bleaching in *P. astreoides* and *O. faveolata*, but not *P. divaricata* (Fig. 3B, F, J). Sensitivity of Mg/Ca is about 0.09–0.16 mmol mol^−1^ per °C [108], [109], [114], and therefore treatment corals were expected to have Mg/Ca ratios that were higher by 0.1–0.2 mmol mol^−1^ compared to control corals at 0 weeks on the reef. Although treatment *P. astreoides* had significantly higher Mg/Ca ratios than control corals at this time point (Fig. 3F), the difference was 0.9 mmol mol^−1^, which is much higher than expected based on temperature dependence alone. Further, Mg/Ca decreased with bleaching in *O. faveolata* instead of the expected increase (Fig. 3J). However, the reliability of Mg/Ca as a SST proxy has been questioned by many studies [98], [108], [112]. Mg/Ca ratios showed similar patterns as calcification rates in both *P. divaricata* and *O. faveolata* and could therefore be mainly controlled by calcification rate. However, they were not significantly correlated in either species (Spearman's *r*  =  0.40, *p*  =  0.10 and *r*  =  0.63, *p*  =  0.13, respectively). They were also not significantly correlated in *P. astreoides* (*r*  =  −0.06, *p*  =  0.84). It is therefore unlikely that Mg/Ca ratios are primarily controlled by calcification rate.

Sensitivity of U/Ca is about 0.03–0.05 µmol mol^−1^ per °C [115] and treatment corals were therefore expected to have U/Ca ratios that are 0.04–0.06 µmol mol^−1^ lower than in control corals at 0 weeks on the reef. However, either no change or the opposite (i.e., increases in U/Ca) was observed. It is therefore likely that seawater temperature is not the primary control on coral U/Ca. Potential salinity [115] and pH effects [115], [116] can be excluded as treatment and control corals were kept in the same seawater (except for temperature). Coral δ^11^B further suggests that the pH of the calcifying fluid did not significantly differ between treatment and control corals (Fig. 2).

Although Ba/Ca ratios are typically used as a proxy for upwelling of nutrient-rich water [108], [117], [118] and river input and flood events [119], [120], in some species Ba/Ca ratios can also be influenced by SST [117], [121]. If that were the case in the present study, treatment corals would be expected to have lower Ba/Ca ratios than control corals at 0 weeks on the reef. However, this was not generally the case. As all corals were kept in the same seawater and location on the reef, both river input and temperature effects are unlikely to have caused the observed pattern.

Interestingly, Ba/Ca ratios of *P. astreoides* were an order of magnitude higher than in either *P. divaricata* or *O. faveolata*, showed high within-treatment variability, and were also much higher than typical flood or upwelling related Ba signals in corals (Fig. 3H) [118], [119], [120]. Considering that all three coral species were exposed to identical environmental conditions except for temperature during the bleaching treatment, this finding is difficult to explain and cannot be related to seawater Ba concentrations. Even in exceptionally high Ba seawater, coral skeletal Ba/Ca concentrations do not exceed 6.5 µmol mol^−1^ [122]. However, similar differences in Ba/Ca between different coral genera have also been observed in another study [123].

One possibility is that particulates and/or organic phases rich in Ba may have become trapped within the skeleton [124], [125]. Due to its encrusting to mounding morphology, sediment settles more easily on *P. astreoides* compared to the other two species (V. Schoepf, pers. observation) and may somehow result in enriched Ba concentrations in the surrounding seawater and/or skeleton. Spawning has also been hypothesized to influence Ba/Ca ratios [125], but the seasonal peak in reproduction occurs in April for *P. astreoides* [126], whereas the corals here were collected in August and September, respectively. Overall, these findings add to the existing literature of anomalously high Ba concentrations in corals, which at present cannot be satisfactorily explained [118], [124], [125], [127].

Overall, trace element data often showed trends that were inconsistent with either changes in seawater temperature or the observed physiological effects. This was in stark contrast to the isotopic data, which showed consistent trends across species, with calcification rate being the main influence on δ^13^C and δ^18^O but not δ^11^B in bleached corals. Given that it is difficult to explain these inconsistencies based on our current understanding of trace elements, these findings demonstrate that the mechanisms of trace element incorporation in bleached corals are poorly understood and that they are likely species-specific. Species-specific differences in the bleaching response of the three coral species studied here [13] may in part be responsible for the observed inconsistencies. For example, *P. divaricata* was least affected by bleaching whereas *P. astreoides* suffered the largest declines of all three species in both symbiont and host performance (Fig. 1A–D) [13]. In contrast, *O. faveolata* showed large health declines initially but was able to recover calcification rates, energy reserves, and endosymbiont densities within 6 weeks (Fig. 1E, F) [13]. Similarly, trace elements showed no significant temperature effects and little within-treatment variability in *P. divaricata* whereas temperature effects were often evident in the other two species (Fig. 3). Although the magnitude and direction of these effects were typically inconsistent with our current knowledge about trace element incorporation and often differed between *P. astreoides* and *O. faveolata* (see above), this could nevertheless indicate that more severely bleached corals may show larger excursions in their trace elements. This study therefore highlights the need to study trace element incorporation under stressful temperatures in a variety of coral species, especially in the context of accompanying physiological information.

### Implications for Paleo-Climate Reconstruction

The present study provides the first experimental evidence that short coral bleaching events do not affect boron isotopes in three Caribbean coral species. This is generally good news because it suggests that past seawater pH conditions can be reconstructed without any conflicting effects introduced by significant physiological changes due to short bleaching events. This is particularly important for massive coral species such as *O. faveolata* or *Porites* sp., which are frequently used for paleo-pH reconstruction. However, at the same time, this study indicates that coral δ^11^B cannot be used as a novel proxy for past bleaching events as suggested by Wei et al. [47]. Similarly, our findings confirm that δ^13^C and δ^18^O cannot be used as bleaching proxies when calcification rates are compromised, demonstrating that there is currently no reliable proxy available to identify past bleaching events. However, future studies are needed to confirm these findings for prolonged coral bleaching events.

Regarding trace elements, Sr/Ca of bleached corals did not consistently record the 1.2°C difference in seawater temperature during the bleaching treatment, or alternatively show a consistent increase due to impaired photosynthesis and calcification. These findings suggest that the mechanisms of Sr/Ca incorporation are not well understood at temperatures that are stressful to corals. Further, the observed changes in Mg/Ca, U/Ca and Ba/Ca due to coral bleaching could not be satisfactorily explained using either temperature dependence or changes in coral physiology. Therefore, it is likely that additional factors influence these geochemical proxies and that these factors are species-specific. It is therefore not recommended to use corals with a history of bleaching for paleo-climate reconstruction using trace elements.
